# Centralized Threshold Key Generation Protocol Based on Shamir Secret Sharing and HMAC Authentication

**DOI:** 10.3390/s22010331

**Published:** 2022-01-03

**Authors:** Shimaa A. Abdel Hakeem, HyungWon Kim

**Affiliations:** 1 Mixed-Signal Integrated System Lab (MSIS), School of Electronics Engineering, Chungbuk National University, Cheongju 28644, Korea; shimaakotb@cbnu.ac.kr; 2 Electronics Research Institute (ERI), El Nozha, Cairo 12622, Egypt

**Keywords:** Shamir secret, key generation, key reconstruction, threshold protocols, vehicular communication, HMAC authentication, centralized protocols, unconditional secure

## Abstract

Many group key management protocols have been proposed to manage key generation and distribution of vehicular communication. However, most of them suffer from high communication and computation costs due to the complex elliptic curve and bilinear pairing cryptography. Many shared secret protocols have been proposed using polynomial evaluation and interpolation to solve the previous complexity issues. This paper proposes an efficient centralized threshold shared secret protocol based on the Shamir secret sharing technique and supporting key authentication using Hashed Message Authentication Code Protocol (HMAC). The proposed protocol allows the group manager to generate a master secret key for a group of n vehicles and split this key into secret shares; each share is distributed securely to every group member. t-of-n vehicles must recombine their secret shares and recover the original secret key. The acceptance of the recovered key is based on the correctness of the received HMAC signature to verify the group manager’s identity and ensure the key confidentiality. The proposed protocol is unconditionally secure and unbreakable using infinite computing power as t, or more than t secret shares are required to reconstruct the key. In contrast, attackers with t−1 secret shares cannot leak any information about the original secret key. Moreover, the proposed protocol reduces the computation cost due to using polynomial evaluation to generate the secret key and interpolation to recover the secret key, which is very simple and lightweight compared with the discrete logarithm computation cost in previous protocols. In addition, utilizing a trusted group manager that broadcasts some public information is important for the registered vehicles to reconstruct the key and eliminate secure channels between vehicles. The proposed protocol reduces the communication cost in terms of transmitted messages between vehicles from 2(t−1) messages in previous shared secret protocols to zero messages. Moreover, it reduces the received messages at vehicles from 2t to two messages. At the same time, it allows vehicles to store only a single secret share compared with other shared secret protocols that require storage of t secret shares. The proposed protocol security level outperforms the other shared secret protocols security, as it supports key authentication and confidentiality using HMAC that prevents attackers from compromising or faking the key.

## 1. Introduction

Recent research introduces many security protocols to improve vehicular group communication security by improving network authentication, availability, integrity, and nonrepudiation. The group key management protocols must provide secure key generation, key distribution, and key updating with a minimum communication overhead [1,2,3]. Many group key generation protocols are proposed in the literature; some are based on the symmetric key generation, and some use the asymmetric key generation [4,5,6]. Most of the previous asymmetric key generation methods suffer from high communication and computation costs, depending on complex elliptic curve mathematics or bilinear pairing [7]. Due to their complexity or security level, these protocols cannot be applied to the new vehicular communication through the 5G networks [8]. The 5G mobile network promises future Machine-to-Machine communications and Vehicle-to-Vehicle communication. Smartphones and traffic monitoring systems have grown exponentially across mobile networks in recent years. The simultaneous access data flow and the enormous number of devices delivering little data have created tremendous security issues [9,10].

Moreover, the generated key in these protocols is stored as one copy at each participant or many copies at different locations, exposing the key security to various attacks. This challenge is addressed by shared secret sharing techniques, allowing for high reliability and trustworthiness. In the previous (t, n) threshold shared secret protocols, a group dealer generates a master secret key for secure group communication and divides the key into parts, then distributes the secret parts to n group members. t of group members collaborate to reconstruct the master secret key by sharing their secrets. The high communication cost due to the share of participants’ secret parts results in network congestion and bandwidth exhaustion.

We discuss the applicability of Shamir secret sharing in group key generation for vehicular networks [11]. Shamir’s secret gives you the ability to take a secret and break it down into smaller pieces. The original secret may be retrieved only after collecting a sufficient number of pieces (t, n). Shamir’s secret sharing technique is information-theoretically secure; however, the scheme is still prevented from being relevant to vehicular communications due to the following known issues:

- The wireless broadcast medium of vehicular communication exposes the security of sharing the secret key components that might put your system at attack risk.
- Collecting t shares to retrieve the master secret key is considered an issue and impractical in vehicular communication with high dynamic nature as vehicles may frequently join and leave the network. This impacts the initial network configuration, requiring a defined list of participants at the initial communication setting.
- The assumption that all group members are honest in a decentralized and untrustworthy network is not possible to consider.
- The individual members can mislead or ignore other participants’ contributions.
- The distribution of each participant’s role in helping others rebuild the final polynomial and recover the group key adds substantial overhead owing to high communication among participants.
- The broadcasting of participants’ shares makes the protocol vulnerable to key recovery attacks since each participant’s secret part is broadcasted in the broadcast channel.
- Anyone with access to the broadcasted shares may reassemble the polynomial and discover the secret key. Moreover, there is a security issue since the private key must first be produced and divided into parts. If the dealer is a bad actor, the private key can be stolen or abused. Therefore, any malicious user can present a forged share without being noticed. It is difficult to detect if the reconstructed secret is invalid.

Due to these issues, in this paper, we introduce a novel centralized group key generation and distribution protocol to address the previously threshold secret sharing issues. It is based on shared secret key splitting and symmetric authentication that utilizes a trusted group manager to broadcast public information that is important only for the registered vehicles. This paper proposes a (t, n) threshold key management protocol using a lightweight polynomial evaluation to generate the key by the trusted manager and polynomial interpolation to recover the key by t-of-n members and verifying the key authenticity using symmetric Hashed Message Authentication Code (HMAC). The group manager prepares several shares using a linear polynomial and securely distributes the secret shares for all group members. Each group member requires at least t shares to reconstruct the key; less than t shares, it is impossible to recover the key. To allow the key reconstruction, the group manager broadcasts specific authenticated public information, including t−1 additional public shares.

We summarize the paper contributions as follows:

- Proposing a centralized threshold group key generation and reconstruction protocol based on Shamir secret share and lightweight polynomial evaluation and interpolation.
- Proposing a lightweight key authentication using the symmetric HMAC protocol ensures the key confidentiality and authenticates the group manager.
- Reducing the communication overhead due to the broadcast of t participants’ shares by including a trusted centralized manager in the key generation process to provide t−1 public points to all authorized members to recover the group key.
- Updating the group key by dynamically generating a new polynomial to prevent the key compromising attacks.
- Adding or removing group members does not affect the validity of the existing shares, which means that the secret shares can be assigned one time at the initial phase while many times apply.
- Reducing the communication cost in transmitting messages between vehicles from 2(*t* − 1) messages in previous shared secret protocols to zero messages.
- Reducing the received messages at vehicles from 2*t* to 2 messages.
- Minimizing the storage to a single secret share compared with other shared secret protocols requiring storing t secret shares.
- Outperforming the security level of other shared secret protocols security by supporting key authentication and confidentiality using HMAC to prevent attackers from compromising or faking the key.

We summarize the contributions and solutions of the proposed protocol over the basic Shamir secret sharing in Table 1.

The rest of this paper is organized as follows. We discuss the related work in Section 2, while Section 3 introduces the preliminary mathematics of Shamir’s secret protocol. We present the proposed key management protocol in Section 4. The numerical examples of the proposed protocol are introduced in Section 5. The security analysis and performance evaluation are presented in Section 6 and Section 7. The conclusions and future work are presented in Section 8.

## 2. Related Work

The threshold (t, n) Shamir’s secret share was developed by Shamir and widely used as a threshold secret sharing technique [11]. Key interpolation or the Lagrange technique is employed in threshold shared secret implementation. Each coefficient in the polynomial represents a portion of the shared master secret. A safe polynomial may be constructed from a collection of points. Once you find a sufficient number of these points, the original polynomial and the original key may be recovered. There is no leaked information about the initial secret via the distribution of secret shares. To recover the original secret, one must collect a sufficient number of shares (t, n). Shamir’s secret sharing uses the polynomial evaluation to generate the key and then applies Lagrange interpolation to reconstruct the original secret. There are security conditions for Shamir’s secret to be safe: (1) knowledge of any t or more than t shares allows reconstruction of the master secret, (2) knowledge of any t shares prevents access to any information about the master secret.

It is safe to say that Shamir’s technique is 100% secure since it meets both of these criteria without making any computational assumptions.

In 1979, two authors—Shamir and Blakley—separately proposed secret sharing [12]. Shamir and Blakley constructed their respective techniques using Lagrange’s interpolation and Blakley’s hyperplane geometry. Mignotte developed a secret sharing technique [13] based on the Chinese Remainder Theorem (CRT), and it was enhanced by AsmuthBlooms [14].

Many academics have expanded Shamir’s [12] method in many contexts. Based on Shamir’s method, Thien and Lin introduced a Secret Image Sharing (SIS) system [15]. If the shares created by Shamir’s technique are disseminated over insecure channels, the shares’ secrecy is challenged, and malicious users may exploit the shares. Because of this, Zhao et al. suggested a solution in [16] for maintaining the confidentiality of shares transmitted across insecure channels. Ulutas et al. [17] developed an extension of the secure key distribution method used in [16] for the safe distribution of shares of medical images. The authors also used Shamir’s framework, which has improved authenticity and confidentiality properties, in addition to the secure key distribution method used in [18]. During the reconstruction of the secret phase, when the shareholders offer their shares, dishonest shareholders might present fake shares, misleading the other honest shareholders. An effective secret sharing method must be able to detect and identify cheaters. They developed a mechanism for detecting and identifying cheaters in Shamir’s secret sharing system. According to [19], redundant shares are employed to detect cheaters when more than t shares are given for reconstruction.

Several secret sharing systems have been presented; each of them may check if the shares distributed by participants are correct under the condition that the secrecy of the shares and the secrecy of the secret are maintained at all times. Harn et al. [20] suggested a verifiable secret sharing method based on the CRT and an extension of Asmuth–Bloom’s scheme [14], based on the CRT.

Liu et al. [21] have presented an efficient technique based on the Asmuth–Bloom scheme. With the expanded Chinese Reminder Theorem, Shamir’s Secret Sharing, and Asmuth–Bloom’s secret sharing, Liu and Chang provided an integrable mechanism for verification in [22], which improves on the verification approach proposed by Harn et al. in [20] by employing a one-way hash function.

The benefits of multiparty computing and function sharing have been investigated [23,24]. The techniques may identify harmful users’ cheating behavior during secret reconstruction, so an honest user cannot be misled. The system might use the Liu et al. technique to distribute several secrets [25]. The approach of Cramer et al. is not unconditionally secure due to the universal hash function. Still, Lin and Harn’s scheme is easily and quickly broken by a simple attack, as shown by Ghodosi [26,27]. Liu et al. detects cheating during secret reconstruction and reduces the user’s share size. Using two polynomials increases the number of calculations. As a consequence, calculation overhead increases.

Meng et al. [28] presented two thresholds changeable secret sharing methods in 2020. One was based on a bivariate polynomial and the other on the mix of bivariate and univariate polynomials. Their approaches use binding values during the hidden rebuilding phase to obtain the threshold changeable characteristic they seek. Based on bivariate polynomial, their technique is unconditionally safe and highly efficient.

Liu et al. [29] proposed a linear threshold secret sharing scheme that combines two of Shamir’s procedures. In [29], the share size comes close to reaching its theoretical lower limit in the case of (k, n) secret sharing with cheating detection.

The cheating detection phase is characterized by the fact that only one honest player can identify cheating from among the other k−1 cheats, resulting in higher detection effectiveness than the prior linear secret sharing methods.

Table 2 summarizes the previously mentioned shared secret protocols’ advantages, disadvantages, and implementation types.

After reviewing some shared secret protocols proposed for group key generation, we noticed a lack of studies to apply the threshold shared secret sharing in vehicular communication.

In this paper, we propose a key generation and distribution solution that depends on the existence of the 5G base stations at short distances, as mentioned in [30]. Currently, vehicular communication is moving towards the cellular infrastructure instead of Dedicated Short Range Communication (DSRC). Thus, the cellular base stations can be utilized as a trusted third party to divide a master secret into several secret shares, then securely distribute the secret shares to the group members. A group key is distributed among participants using a threshold secret sharing technique in 5G networks scenarios. Afterward, the key is recovered only when it is required, with no need to store the secret key at the participant’s side for a long time. It has also been recommended for 5G networks, which are vulnerable to being tapped, that data be shared in secret to make the eavesdropper’s mission more difficult. It is possible to increase the level of security in these situations by continually modifying the structure of the shares. Therefore, implementing dynamic threshold secret sharing in 5G networks is important.

## 3. Preliminary

Polynomials in Shamir’s secret sharing are generally of the following form:

(1) $$f\left(x\right)=a_{0}+a_{1}x^{1}+a_{2}x^{2}+a_{3}x^{3}+\ldots +a_{t-1}x^{t-1}\;mod\left(p\right)$$

Participants’ shares are represented as coefficients in the polynomial, chosen at random by the dealer. The value of the shared master secret can be broken into parts; this is the value of the free coefficient $a_{0}$, the degree of polynomials is t−1, indicating that the number of coefficients is t always (as it includes the free coefficient). Lagrange interpolation for f(x) recovery: The y-coordinate of a point on the polynomial is obtained by evaluating f(x). This means that a single point on the polynomial is defined by (x, y=f(x)). If Lagrange interpolation is utilized, just t points on the degree t−1 polynomial are required for the reconstruction. Then, we have the following set of t−1 shares: $\left(x_{0},\;y_{0}\right),\;\left(x_{1},\;y_{1}\right),\;\ldots ,\;\left(x_{t-1},\;y_{t-1}\right)$. We can construct the following type of t Lagrange base polynomials:

(2) $$l_{j}\left(x\right)=\prod_{i=0,i\neq j}^{t-1}\frac{x-x_{i}}{x_{j}-x_{i}}$$

Using these Lagrange base polynomials, we can reconstruct f(x):

(3) $$f\left(x\right)=\sum_{j=0}^{t-1}\;y_{j}l_{j}\left(x\right)mod\left(p\right)$$

**Figure 1:**
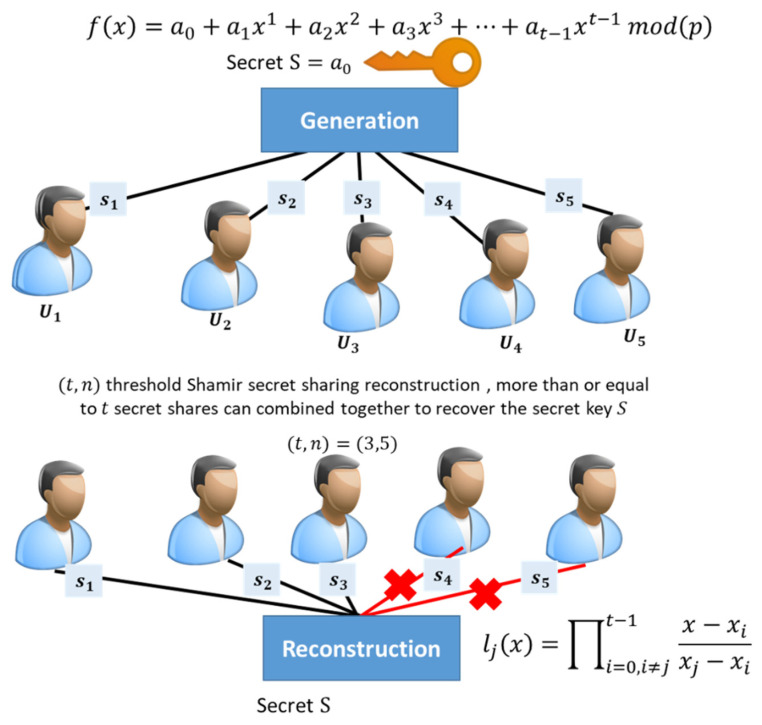
The threshold Shamir secret sharing key generation and reconstruction.

Because we only care about the value of f (0), which is the same as the free coefficient of f(x), the calculation can be shortened to

(4) $$f\left(0\right)=\sum_{j=0}^{t-1}\;(y_{j}\;\prod_{i=0,i\neq j}^{t-1}\frac{x_{i}}{x_{i}-x_{j}})mod\left(p\right)$$

where f (0) represents the original secret key. (t,n) threshold Shamir secret sharing example is illustrated in Figure 1.

## 4. The Proposed Protocol

In this section, we present the proposed key generation protocol. One trustworthy entity, the Group Manager (G.M), selects the master key for the group and then splits it into different secret pieces that can be distributed securely to all participants. To subscribe to the key distribution service, each user must first register with the G.M. The G.M maintains a list of all registered users and removes those prevented from receiving group communications. We introduce the proposed protocol steps in the following subsections.

### 4.1. Network System Model

There are two types of security protocols, distributed key management protocols and centralized key management protocols. In distributed key management, each vehicle has to compute the group key in real-time and share the key management burden and generation computation and communication cost to each member in the group. Thus, it spreads the key management burden throughout the group, increasing security and fault tolerance in integrity and secrecy. The centralized protocols in which the trusted third party can manage the key generation, the distribution, and the key updating process. These approaches reduce the overhead on the joined vehicles that generate the key or distribute it.

This paper proposes a centralized key management protocol where the B.Ss of 5G networks are considered a trusted third party to generate the secret keys, distribute them through secure channels, and update it if needed. 5G is the next generation of mobile radio technology, which will allow for much higher data rates and lower latency [31]. 5G devices are expected to be numerous, resulting in a significant increase in traffic. As a result, better cell deployment is urgently required. A large number of base stations are thus needed to provide local security and privacy management services. A group of vehicles covered by the same Base Station (B.S) joins the same group communication.

**Figure 2:**
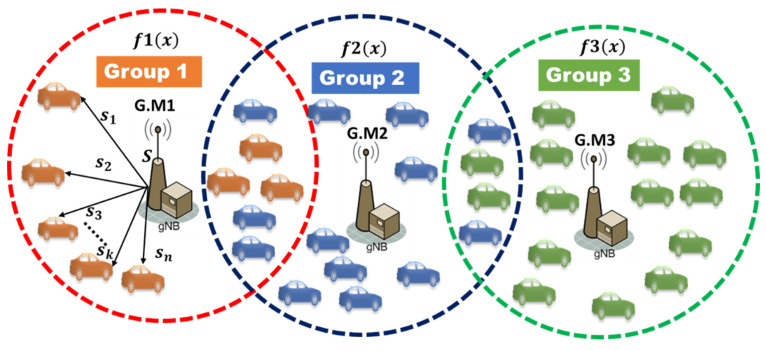
The proposed network system model.

This paper focuses on the inter-group communications between vehicles attached to the same B.S. We assume that only vehicles can communicate with the nearest vehicles in the same communication range. Each B.S has unique parameters to assign the secret shares for the participants and generate one different master secret key per group. Vehicles attached to the same base station have the same base station identifier and same group identifier. The network system model is divided into groups; each group is covered by one trusted B.S that can choose a safe polynomial f(x) to evaluate it and generate the master secret key S, then split it into n secret shares {$s_{1},s_{2},s_{3},\ldots ,s_{n}$}, then distribute it securely for participants as shown in Figure 2.

### 4.2. Vehicles Registration

All vehicles initially are authenticated to their B.Ss through the 5G-AKA protocol that supports the primary authentication for vehicles against the 5G core network; for more information, readers can refer to [32]. After primary authentication is complete, each vehicle is assigned a specific private key. Using this pre-shared key, the vehicle and the 5G network can authenticate each other and establish a secure communication channel.

After the 5G-AKA primary authentication is complete, the vehicles communicate the B.S through a secure channel using the previously shared private key. This paper proposes a centralized interactive key generation protocol to minimize the dependency on the other vehicles to reconstruct the master key parts in previous related Shamir sharing protocols. We allow the base station to represent a Key Distribution Center (KDC) for the joined vehicles. Vehicles joined to the same base station form a group, as one vehicle (initiator) sends a group request to the base station to start a group communication. After all joined vehicles are registered at the B.S, the B.S chooses a master secret key for the vehicles n. B.S splits the secret S into parts according to Shamir’s secret sharing scheme; then it distributes a secret S among n vehicles $\left\{V_{1},\;V_{2},\;V_{3},\;\ldots ,V_{n}\right\}$.

### 4.3. Key Generation and Distribution

In this paper, we utilize an optimized threshold secret sharing scheme that allows a group of vehicles to share one secret key securely without transferring this key individually to all participants in an encrypted way.

The proposed centralized group key protocol consists of three phases, the key generation phase, the key authentication phase, and the key reconstruction. We discuss all three phases in the following subsections. 

#### 4.3.1. Key Generation

The base station picks a random polynomial f(x) of degree (t−1), then it calculates the secret key S and splits it into (n) shares $\left\{Share_{i}\;\left(s_{i}\right)=\left(x_{i},\;f\left(x_{i}\right)\right)\right\}$ and a prime number (p). After the base station generates the secret shares for each vehicle joined the group, it securely distributes the shares to the vehicles through a secure channel. Then, each vehicle has only one valid secret part from the master secret and waiting for (t−1) parts to reconstruct the group master secret key S. The base station starts broadcasting (t−1) random distinct points from the polynomial as $x_{i}$ ≠ 0 for all the group members that can be important only for the registered authorized vehicles.

**Figure 3:**
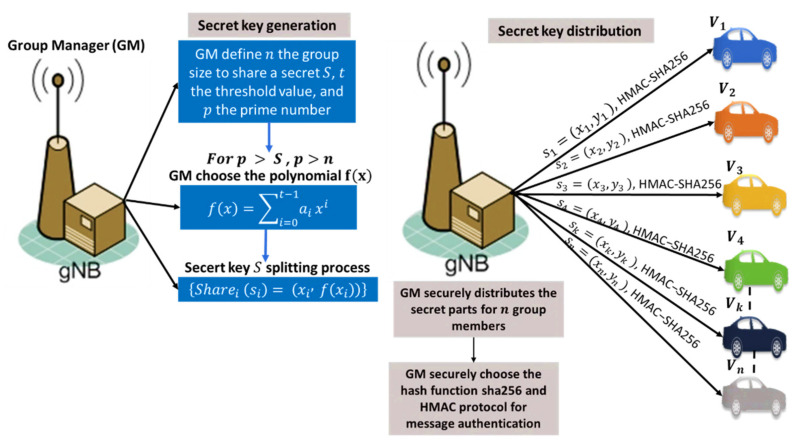
The proposed key generation and distribution process.

To split the secret into shares, the B.S creates a polynomial *f*(*x*) of degree *t* − 1 using Equation (1) and a constant term *a*_0_ that represents the secret coefficient, where *p* is a prime number selected based on the level of security needed for the secret, and the constant term $a_{0}$ is the secret S. Higher values of p result in greater security, and the secret S is always less than the prime number p and typically more than n. The t−1 secret share are integer values represented by the coefficients $a_{1},\;a_{2},\;\ldots ,\;a_{t-1}$ of (*x*) and chosen such that $a_{i}\;\in \;\left[0,\;p\right]$ for all i. Using the simplified integer arithmetic method to generate the shared secret key results in some security problems. Every participant gains a great deal of information about the secret key with every share $\left(s_{i}\right)$, which exploits a security attack in respect of the polynomial order and gains a great deal of information about all valid points over the polynomial. We use polynomial operations over finite field arithmetic to reduce this security attack, making it hard for participants or attackers to define the used polynomial in a generation. The polynomial curve over a finite field is disordered and disorganized, unlike the conventional curves that make it hard to discover new valid paths between points that can be considered secret shares of the master secret key. Choosing the finite field size, while p>n, p>s, the bigger the p size, the more challenging it is to solve the equation and find the secret shares. Then, the calculation of the secret share is $\left(x_{i},\;f\left(x_{i}\right)\mathrm{mod}\;p\right)\}$ instead of $(x_{i},\;f\left(x_{i}\right).$ Finite field arithmetic enhances the security level and provides more potential to find the secret shares. We summarize the key generation process performed by the G.M as follows:

- Create a group of size n to share a secret S.
- Choose the required number t of participants to recover the key (threshold value to reconstruct the master secret key).
- Select the prime number p to define the level of security needed over the finite field $F_{p}$ of size p elements and p>S, p>n.
- Select minimum number m for the key-value.
- Choose the key-value between m and p−1.
- Build a random polynomial f(x) using the parameters (n, t,p, S).
- Split the secret S into distinct points on the polynomial f(x).
- Distribute the secret shares securely $\left\{Share_{i}\;\left(s_{i}\right)=\left(x_{i},\;f\left(x_{i}\right)\right)\right\}$ to the group members.
- Distribute the selected one-way hash function (SHA256) securely to be used with the HMAC algorithm to verify secret shares and secret keys. The key generation process is shown in Figure 3 and illustrated in Algorithm 1.

**Algorithm 1**. Key generation based on Shamir secret.**Input:**  ●List of the all registered vehicles $\left\{V_{i},\;V_{2},\;V_{3},\;\ldots ,V_{n}\right\}$ in the group sent by initiator vehicle asking for a group key distribution service.**Output:**  1.G.M randomly picks a polynomial f(x) of degree (t−1):  $f\left(x\right)=a_{0}+a_{1}x^{1}+a_{2}x^{2}+a_{3}x^{3}+\ldots +a_{t-1}x^{t-1}\;mod\left(p\right)$ in which the secret $S=a_{0}=f\left(0\right),$ and all coefficients $a_{0};a_{1};\ldots a_{t-1}$ are in the finite field $F_{p}$ of size p elements and p>S, p>n.  2.G.M. divides the secret key S into n parts, then calculates shares: $\left\{Share_{i}\;\left(s_{i}\right)=\left(x_{i},\;f\left(x_{i}\right)\right)\right\}\;$ = $\left(x_{i},f\left(i\right)\;\left(mod\;p\right)\right)$ for i=1; 2…n.  3.G.M. securely distributes the (n) shares $s_{i}$ to the (n) vehicles.  4.G.M. generates message m that consists of (t−1) additional public points $P_{i}$ on the polynomial f(x), for i=1;2; 3;…; t−1; a list of group members $n=\left\{V_{1},\;V_{2},\;V_{3},\;\ldots ,V_{n}\right\}$, the prim number p; and the threshold t.  5.G.M. generates an HMAC signature over message m using the secret key S: $HMAC-SHA256_{\mathrm{secret}\;\mathrm{key}\;S\;}\;\left(m\right)$, then broadcasts m attached to the signature HMAC.

#### 4.3.2. Key Authentication

**Figure 4:**
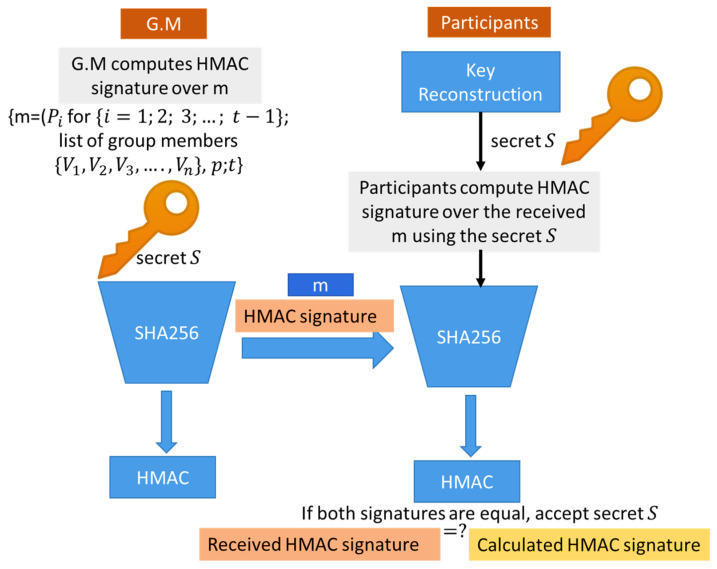
The proposed key authentication process using HMAC.

In contrast to previous key management protocols that use very complex authentication methods such as elliptic curve operations and bilinear pairing authentication, we utilize the fast and straightforward HMAC-SHA256 protocol to ensure message authentication and integrity in the proposed key generation method. HMAC protocol allows the sender and receiver to share a shared secret key to calculate a Message Authentication Code signature over the transmitted message. At the sender side, the G.M generates an HMAC signature using the generated master secret key S and the one-way hash function SHA256. The G.M hashing the message m using SHA256 then calculates the HMAC signature using the secret key S. The G.M transmits the key generation message m that contains t−1 public points attached to the HMAC signature to allow the participants to authenticate the G.M and reconstruct the master secret S. Moreover, it helps the participants verify the secret key secrecy and authenticate the G.M. The HMAC-SHA256 is a straightforward, low overhead authentication protocol that ensures message authentication and integrity [33]. The HMAC authentication is shown in Figure 4.

#### 4.3.3. Key Reconstruction

In previous methods, all participants must share their secret parts to allow others to construct the master secret key, which exposes the security of the key to key compromising attacks. This paper enables the G.M to broadcast unique public points from the polynomial to allow each registered vehicle to recover the master secret key. We reduced the communication overhead between vehicles to transfer and exchange their secret shares. We also enhanced the security level by including the G.M as a trusted third party to verify all participants and authorize their access.

**Figure 5:**
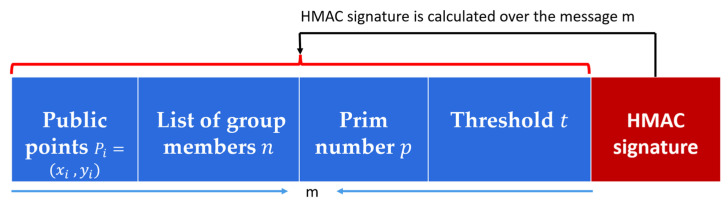
The proposed key reconstruction message structure.

**Figure 6:**
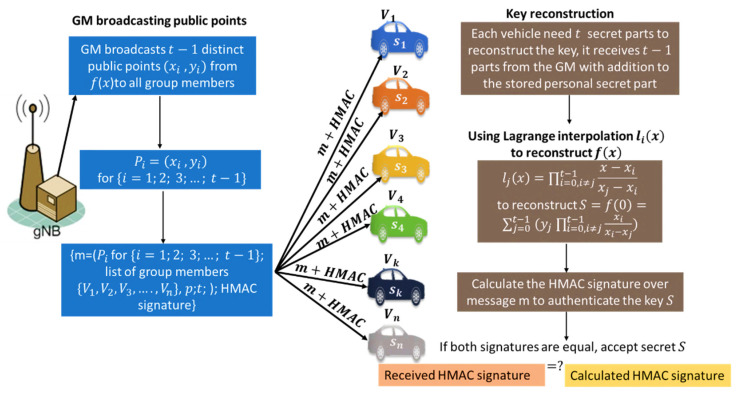
The proposed key reconstruction process.

- G.M broadcasts t−1 distinct public points from *f*(*x*), where $P_{i}=\left(x_{i},\;y_{i}\right)$ for  {i=1;2; 3;…; t−1}.
- The key reconstruction message m broadcasted by the G.M consists of m = (The public points ($P_{i}$ for {i=1;2; 3;…; t−1}; a list of group members n = $\left\{V_{1},\;V_{2},\;V_{3},\;\ldots ,V_{n}\right\}$; the prim number p; the threshold t); attached to the HMAC signature. The key reconstruction message structure is shown in Figure 5.
- HMAC signature represents a hashed value over the received message m using the reconstructed master secret S.
- Each group member reconstructs the polynomial f(x) based on its secret share $s_{i}$ and the broadcasted public points $P_{i}$ from the G.M using Lagrange interpolation $l_{i}\left(x\right)$ That is defined by Equation (2). Equations (2)~(4) are used to reconstruct the polynomial f(x) and calculate the free coefficient f(0) that represents the master secret S.
- After reconstructing the secret, each participant has to authenticate the G.M to accept or reject the key. The authentication process is performed using the HMAC-SHA256 protocol by calculating the hash value of the received message m using SHA256 and then calculating the message authentication code over the m using HMAC and the constructed key S to authenticate the G.M as shown in Figure 4.
- Each participant compares the calculated HMAC signature, and the received HMAC signature, then only accepts S if both signatures are equal. This can prove the key secrecy and confidentiality.
- Each participant at least requires t points to reconstruct the secret S, and t should be less than or equal to (n), i.e., 1 ≤ t ≤ n.
- Each participant received a broadcasted message from the G.M that consisted of the (t, n) public points, only registered vehicles can recover the key (S) using Equation (4) of Lagrange to calculate f (0) that represents the group secret key. Each vehicle has $Share_{i}\;\left(s_{i}\right)$ and (S) stored at its hardware security module for secure group communication. The key reconstruction process is shown in Figure 6 and illustrated in Algorithm 2. Figure 6 describes the key reconstruction process that contains two basic steps: the group manager performs the first step and is represented by the blue boxes at the left. This step has required broadcasting some public information from the manager to all participant vehicles. At the same time, the second step represents the reconstruction process at vehicles. It is represented by brown boxes to describe the formulas used by the participant to recover the secret key based on the received information from the group manager.

**Algorithm 2**. Key Reconstruction based on Shamir secret and HMAC.
**Input:**
  ●Each participant receives m = {($P_{i}$ for {i=1;2; 3;…; t−1}; list of group members $\left\{V_{1},\;V_{2},\;V_{3},\;\ldots ,V_{n}\right\}$, the prim number p; the threshold t), HMAC signature} from the G.M.  ●Each participant stores a valid single secret share $s_{i}$.
**Output:**
  1.Each member recovers the secret S using Lagrange interpolation $l_{i}\left(x\right)$, t−1 public points and his share $s_{i}$, as each member need at least t points to reconstruct the polynomial f(x) and recover the group shared secret key $S=a_{0}=f\left(0\right)$.  2.Participant vehicles calculate the hash value of the received message m using SHA256 and the HMAC protocol to generate a signature over the message m using the constructed secret S: $HMAC-SHA256_{\mathrm{secret}\;\mathrm{key}\;S\;}\left(m\right)$.  3.Then, it compares the received HMAC signature from the G.M and the calculated HMAC at vehicles to authenticate that message is broadcasted by authorized G.M.  4.If the calculated HMAC at vehicles is equal to the received HMAC from G.M, the reconstructed key at the vehicles is correct and valid for future group communications.



## 5. Numerical Examples

Given t distinct points $\left(x_{i},\;y_{i}\right)$ of the form $\left(x_{i},\;f\left(x_{i}\right)\right)$, where f(x) is a polynomial of degree less than t, then f(x) is determined by

(5) $$f\left(x\right)=\sum_{i=1}^{t}y_{i}\;\prod \frac{x-x_{i}}{x_{i}-x_{j}}$$

Shamir’s scheme is defined for a secret S ∈ $F_{p}$ with p prime, by setting $a_{0}$ = S, and choosing $a_{1},\;\ldots ,\;a_{t-1}$ at random in $F_{p}$. The trusted party (G.M) computes f(i), where

(6) $$f\left(x\right)=\sum_{i=0}^{t-1}a_{i}x^{i}$$

For all 1 ≤ i ≤ n. The shares $\left(x_{i},\;f\left(x_{i}\right)\right)$ are distributed to the n distinct participants. Since the Secret is the constant term $a_{0}\;$ = S=f(0), the Secret is recovered from any t shares $\left(x_{i},\;f\left(x_{i}\right)\right)$, for I ⊂ {1, …, n} by

(7) $$f\left(0\right)=\sum_{j=0}^{t-1}\;(y_{j}\;\prod_{i=0,i\neq j}^{t-1}\frac{x_{i}}{x_{i}-x_{j}})$$

### 5.1. Key Generation Example

The G.M randomly chooses a polynomial that satisfies the secret-sharing conditions to generate the secret shares and distributes them to all participants. After generating the shares, the G.M also generates random public distinct points on the polynomial to allow the participants to recover and reconstruct the master secret key. All arithmetic operations are performed over a finite field *F_p_*. The prime number p must be greater than the secret key and the number of participants. All generated shares have the same size as the master secret key. This section provides a numerical example of the (3,8) threshold key generation process. The G.M chooses the secret key randomly to be 12, and the polynomial coefficients $a_{1}$ and $a_{2}$ are 10 and 20, respectively, so the polynomial can be formulated as follows:

For n = 8, for t = 3 and for prime number p = 23, the following operations are required:

(8) $$f\left(x\right)=12+10x+20x^{2}$$

The generated shares should be in the form $\left(x_{i},\;f\left(x_{i}\right)\right)$, so the G.M can generate as many distinct points over the polynomial:

(9) $$Share_{1}:\left(1,f\left(1\right)\right)=12+10\left(1\right)+20(1^{2})=\left(1,42mod23\right)=\left(1,19\right)\;Share_{2}:\left(2,f\left(2\right)\right)=12+10\left(2\right)+20(2^{2})=\left(2,112mod23\right)=\left(2,20\right)\;Share_{3}:\left(3,f\left(3\right)\right)=12+10\left(3\right)+20(3^{2})=\left(3,222mod23\right)=\left(3,15\right)\;Share_{4}:\left(4,f\left(4\right)\right)=12+10\left(4\right)+20(4^{2})=\left(4,372mod23\right)=\left(4,4\right)\;\;Share_{5}:\left(5,f\left(5\right)\right)=12+10\left(5\right)+20(5^{2})=\left(5,562mod23\right)=\left(5,10\right)\;Share_{6}:\left(6,f\left(6\right)\right)=12+10\left(6\right)+20(6^{2})=\left(6,792mod23\right)=\left(6,10\right)\;Share_{7}:\left(7,f\left(7\right)\right)=12+10\left(7\right)+20(7^{2})=\left(7,1062mod23\right)=\left(7,4\right)\;Share_{8}:\left(8,f\left(8\right)\right)=12+10\left(8\right)+20(8^{2})=\left(8,1372mod23\right)=\left(8,15\right)$$

(10) $$Share_{9}=\left(9,f\left(9\right)\right)=12+10\left(9\right)+20(9^{2})=\left(9,1722mod23\right)=\left(9,20\right)\;Share_{10}=\left(10,f\left(10\right)\right)=12+10\left(10\right)+20(10^{2})=\left(10,2112mod23\right)=\left(10,19\right)$$

For n=8, shares are distributed securely to the eight participants. At the same time, other unique points over the polynomial with size t−1 are generated to help the participants recover the master secret S without frequent communication.

**Figure 7:**
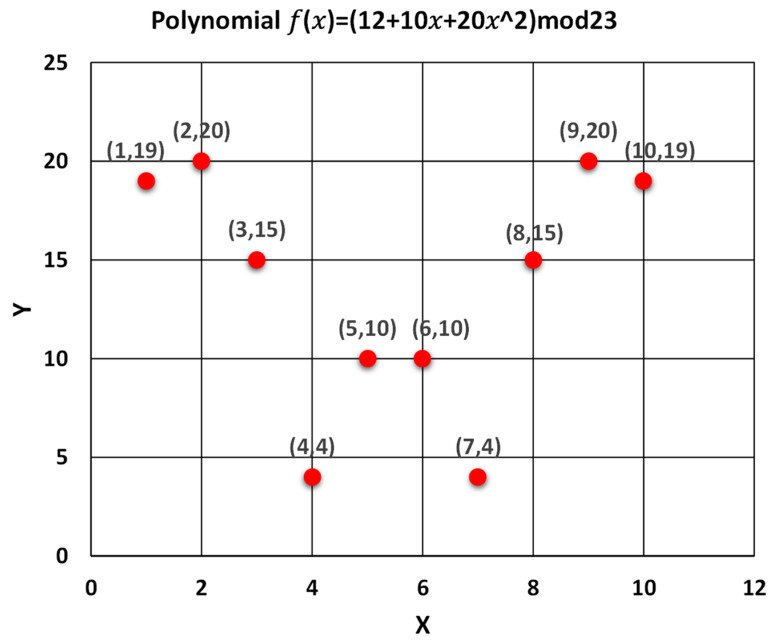
The graphical representation of the generated secret shares over a finite field $F_{23}$.

Each participant now has two shares representing the broadcasted public points from the G.M and one personal secret share that is securely distributed with every participant in the group. The random generated public points for t=3, so the number of the required public points is t−1. Figure 7 shows the graphical representation of the generated secret shares from the polynomial function $f\left(x\right)=12+10x+20x^{2}$ over $\;F_{23}$. Table 3 represents the secret shares coordinates $\left(x_{i},\;f\left(x_{i}\right)\right)$ over the polynomial f(x).

### 5.2. Key Reconstruction Example

According to the mentioned example in the previous section, we allow the vehicles to reconstruct the master secret key S. Using any t shares $\left(x_{1},\;y_{1}\right),\ldots \;,\left(x_{t},\;y_{t}\right)$ and from Equations (2)~(4), each vehicle can calculate the master secret key $a_{0}=S=f\left(0\right).$ For example, we pick the following shares (1,f(1)), (2,f(2)), and(3,f(3)) and, using Equation (2), we derive the Lagrange equations:

(11) $$l_{j}\left(x\right)=\prod_{i=0,i\neq j}^{t-1}\frac{x-x_{i}}{x_{j}-x_{i}}\;l_{0}\left(x\right)=\frac{\left(x-2\right)\left(x-3\right)}{\left(1-2\right)\left(1-3\right)}\;=(2^{-1}).\left(x-2\right)\left(x-3\right)mod23\;\;\;\;\;\;\;\;\;\;\;\;\;\;\;\;\;\;\;\;\;\;\;\;\;\;\;\;\;\;\;\;\;\;\;\;\;\;\;\;\;\;\;\;\;\;\;\;\;\;\;\;\;\;\;\;\;\;\;\;\;\;\;\;\;\;\;\;\;\;\;\;\;\;\;\;\;\;\;\;\;\;\;=12\left(x-2\right)\left(x-3\right)mod23\;\;\;\;\;\;\;\;\;\;\;\;\;\;\;\;\;\;\;\;\;\;\;\;\;\;\;\;\;\;\;\;\;\;\;\;\;\;\;\;\;\;\;\;\;\;\;\;\;\;\;\;\;\;\;\;\;\;\;\;\;\;\;\;\;\;\;\;\;\;\;\;\;\;\;\;\;\;\;=12(x^{2}-5x+6)mod23\;\;\;\;\;\;\;\;\;\;\;\;\;\;\;\;\;\;\;\;\;\;\;\;\;\;\;\;\;\;\;\;\;\;\;\;\;\;\;\;\;\;\;\;\;\;\;\;\;\;\;\;\;\;\;\;\;\;\;\;\;\;\;\;\;=12x^{2}+9x+3$$

(12) $$l_{1}\left(x\right)=\frac{\left(x-1\right)\left(x-3\right)}{\left(2-1\right)\left(2-3\right)}=(-1^{-1}).\left(x-1\right)\left(x-3\right)mod23\;\;\;\;\;\;\;\;\;\;\;\;\;\;\;\;\;\;\;\;\;\;\;\;\;\;\;\;\;\;\;\;\;=22\left(x-1\right)\left(x-3\right)mod23\;\;\;\;\;\;\;\;\;\;\;\;\;\;\;\;\;\;\;\;\;\;\;\;\;\;\;\;\;\;=22(x^{2}-4x+3)mod23\;\;\;\;\;\;\;\;\;\;\;\;\;\;\;\;\;=22x^{2}+4x+20$$

(13) $$l_{2}\left(x\right)=\frac{\left(x-1\right)\left(x-2\right)}{\left(3-1\right)\left(3-2\right)}\;=(2^{-1}).\left(x-1\right)\left(x-2\right)mod23\;\;\;\;\;\;\;\;\;\;\;\;\;\;\;\;\;\;\;\;\;\;\;\;\;\;\;\;\;\;\;\;\;\;\;\;\;\;\;\;\;\;\;\;\;\;\;\;\;\;\;\;\;\;\;\;\;\;\;\;\;\;\;\;\;\;\;\;\;\;\;\;\;\;\;\;\;\;\;\;\;\;\;=12\left(x-1\right)\left(x-2\right)mod23\;\;\;\;\;\;\;\;\;\;\;\;\;\;\;\;\;\;\;\;\;\;\;\;\;\;\;\;\;\;\;\;\;\;\;\;\;\;\;\;\;\;\;\;\;\;\;\;\;\;\;\;\;\;\;\;\;\;\;\;\;\;\;\;\;\;\;\;\;\;\;\;\;\;\;\;\;\;\;=12(x^{2}-3x+2)mod23\;\;\;\;\;\;\;\;\;\;\;\;\;\;\;\;\;\;\;\;\;\;\;\;\;\;\;\;\;\;\;\;\;\;\;\;\;\;\;\;\;\;\;\;\;\;\;\;\;\;\;\;\;\;\;\;\;\;\;\;\;\;\;\;\;\;=12x^{2}+10x+1$$

Therefore, the polynomial ′f(x) is reconstructed using Equation (3) as follows:

(14) $$\begin{array}{c} \prime f\left(x\right)=\overset{t-1}{\underset{j=0}{\sum }}\;y_{j}l_{j}\left(x\right)mod\left(p\right)\\ \;\;\;\;\;\;\;\;\;=f\left(1\right)l_{0}\left(x\right)mod23+f\left(2\right)l_{1}\left(x\right)mod23+f\left(3\right)l_{2}\left(x\right)mod23\\ \;\;\;\;\;\;\;\;\;=19\left(12x^{2}+9x+3\right)mod23+20\left(22x^{2}+4x+20\right)mod23+15\left(12x^{2}+10x+1\right)mod23\\ \;\;\;\;\;\;\;\;\;=12+10x+20x^{2}\; \end{array}$$

We observe from previous calculations that reconstructed polynomial ′f(x) in Equation (14) matches f(x) in Equation (8) and the free coefficient is 12, which represents the master secret key S=f(0) that generated by the G.M. All previous calculations were performed over a finite field $F_{23}$ using the modular addition, modular multiplication, and the modular multiplicative inverse.

For example, we pick other different shares (4,f(4)), (5,f(5)), and(6,f(6)) and, using Equation (2), we derive the Lagrange equations:

(15) $$l_{0}\left(x\right)=\frac{\left(x-5\right)\left(x-6\right)}{\left(4-5\right)\left(4-6\right)}=(2^{-1}).\left(x-5\right)\left(x-6\right)mod23\;\;\;\;\;\;\;\;\;\;\;\;\;\;\;\;\;\;\;\;\;\;\;\;\;\;\;\;\;\;\;\;\;\;\;\;=12(x^{2}-11x+30)mod23\;\;\;\;\;\;\;\;\;\;\;\;\;\;\;\;\;\;\;=12x^{2}+6x+15$$

(16) $$l_{1}\left(x\right)=\frac{\left(x-4\right)\left(x-6\right)}{\left(5-4\right)\left(5-6\right)}=(-1^{-1}).\left(x-4\right)\left(x-6\right)mod23\;\;\;\;\;\;\;\;\;\;\;\;\;\;\;\;\;\;\;\;\;\;\;\;\;\;\;\;\;\;\;\;\;=22(x^{2}-10x+24)mod23\;\;\;\;\;\;\;\;\;\;\;\;\;\;\;\;\;\;\;=22x^{2}+10x+22$$

(17) $$l_{2}\left(x\right)=\frac{\left(x-4\right)\left(x-5\right)}{\left(6-4\right)\left(6-5\right)}\;=(2^{-1}).\left(x-4\right)\left(x-5\right)mod23\;\;\;\;\;\;\;\;\;\;\;\;\;\;\;\;\;\;\;\;\;\;\;\;\;\;\;\;\;\;\;\;\;\;\;\;=12(x^{2}-9x+20)mod23\;\;\;\;\;\;\;\;\;\;\;\;\;\;\;\;\;\;\;\;\;=12x^{2}+7x+10$$

Therefore, the polynomial ′f(x) is reconstructed using Equation (3) as follows:

(18) $$\begin{array}{c} \prime f\left(x\right)=\sum_{j=0}^{t-1}\;y_{j}l_{j}\left(x\right)mod\left(p\right)\\ \;\;\;\;\;\;\;\;\;\;=f\left(1\right)l_{0}\left(x\right)mod23+f\left(2\right)l_{1}\left(x\right)mod23+f\left(3\right)l_{2}\left(x\right)mod23\\ \;\;\;\;\;\;\;\;\;\;=4\left(12x^{2}+6x+15\right)mod23+10\left(22x^{2}+10x+22\right)mod23+10\left(12x^{2}+7x+10\right)mod23\\ \;\;\;\;\;\;\;\;\;\;=\{(2x^{2}+x+14)+(13x^{2}+8x+13)+(5x^{2}+x+8)\}mod23\\ \;\;\;\;\;\;\;\;\;\;=12+10x+20x^{2} \end{array}$$

From previous calculations, we prove that different shares over the same polynomial f(x) result in the same free coefficient representing the same secret key for the group. Any generated points over the polynomial f(x) that are equal to t or greater than t can reconstruct the secret key. The participants do not need to share their secret parts through broadcasting or unicast channel. The centralized group manager can broadcast the required number of public points that help only the registered participants recover the master secret key.

## 6. Security Analysis

This paper proposes a key management protocol by utilizing a combination of polynomials and secret key splitting. The proposed protocol introduces authenticated lightweight key generation and key reconstruction protocol. This section provides a detailed security analysis by introducing the resistance of the protocol against key recovery attacks and introducing the satisfying of some security properties.

### 6.1. Resistance to Key Recovery Attacks

Splitting one master secret key between n vehicles can prevent the distribution of a single master key through traditional key distribution protocols. Our proposed method provides a unique and deterministic key distribution based on Shamir secret sharing by dividing one master key into pieces and distributing these pieces securely to all participants. Only the registered participants can reconstruct the group secret key based on some public values broadcasted by the group manager. The dependence on the existence of t vehicles to reconstruct the key makes it impractical, especially if some vehicles are revoked. The share of each vehicle secret piece in the broadcast channel makes it exposed for key recovery attacks. Any attacker can gather the broadcasted shares, reconstruct the polynomial, and find the secret key. We introduce a centralized interactive solution that utilizes the base station to broadcast t−1 secret shares for only registered vehicles at B.S. If an attacker receives the broadcast message from the base station, he cannot recover the key as reconstructing the key needs at least t secret pieces; based on the received information, the attacker constructs a different polynomial with a different free coefficient $a_{0}=S$.

Each authorized vehicle shares a secret share with the base station through a secure channel at the registration phase. In addition to the public shares broadcasted by the base station, this personal share helps each vehicle recover the group key. Including the trusted third party (G.M) increases the security level and reduces the communication overhead among vehicles. The proposed protocol can resist the key recovery attacks due to shares broadcasting during the key reconstruction. There is no need for the participant to contact each other for share gathering to reconstruct the key as the public information from the G.M allows them to recover the key.

### 6.2. Key Confidentiality

Only the trusted group manager knows the master secret key and the polynomial generated in the proposed protocol. After the vehicles register and authenticate themselves to the G.M, they can participate in the key generation process; otherwise, the G.M prevents unauthorized vehicles from participating. If an attacker received the broadcasted message from the G.M, it still has difficulty joining the network as every participant has a unique secret part at the beginning of communication. Only registered authorized vehicles can retrieve the master secret and communicate with other vehicles through secure group communication.

### 6.3. Key Authentication

Verifiable secret sharing is a well-known problem in prior Shamir’s secret sharing schemes, and it has been addressed in this new implementation. Verifiable secret sharing ensures that shareholders are truthful and not submitting fake shares. However, in our proposed protocol, participants do not share their secret shares to help each other reconstruct the key. All participants receive the same authenticated public points from the trusted group manager (B.S). The broadcasted public points from the B.S are authenticated by calculating a message authentication code using the HMAC-SHA256 protocol. The G.M hashes the key reconstructed message m using SHA256 and generates the HMAC signature using the shared secret key S. The G.M attaches the HMAC signature value over message m to allow the authentication of the group manager and support the key authentication. Only a valid HMAC signature enables the acceptance of the reconstructed key S.

All participants calculate the HMAC signature over the received message m to ensure the authenticity of the G.M and verify the shared secret secrecy. The participants compare the calculated HMAC and received HMAC to accept or reject the key that can enhance the security level, improve the key authentication and prevent the acceptance of fake secret keys from unauthorized parties. No unproven theories are involved in the proposed protocol, and no information is disclosed by distributing shares. In contrast, most public-key cryptosystems exploit known difficulties (discrete logarithm issues, integer factorization) to assure security.

The proposed HMAC protocol ensures key authentication and supports perfect key secrecy while reducing computation overhead.

### 6.4. Dynamic Key Updating

When threshold t shares required to recover the original secret key are kept fixed and constant value, vehicles can be dynamically added to the network or deleted from it without affecting the key reconstruction process. Only the group manager can update the master secret key and generate a new polynomial function with unique coefficients as participants shared secrets. Moreover, the security can be enhanced without changing the original secret, but by only changing the polynomial occasionally (while keeping the same free coefficient f (0) that represents the original secret key) and constructing new public points and secret shares for the participants. The G.M also can generate a new secret key for the group and share new secret parts with each participant according to the previous key updating policy agreed between the group manager and members.

### 6.5. Threshold Key Reconstruction

The proposed protocol solves the problem of key reconstruction that requires at least t secret parts to reconstruct the master group key. In previous Shamir secret sharing schemes, the participants had to communicate to share their secret shares. Each participant needs at least t parts to recover the master key. In this paper, we allow the group manager to broadcast only t−1 valid points over the polynomial f(x), for all registerd vehciles.

In addition to each participant’s secret share, all registered vehicles reconstruct the group shared secret key. In contrast to the previous shared secret protocols that require n members to recover the key, our proposed protocol allows only t members to reconstruct the key. The proposed threshold protocol is significant for different vehicular scenarios in which many vehicles can join or leave dynamically.

### 6.6. Security Analysis Comparison

To our knowledge, our protocol is the first shared secret scheme based on Shamir secret sharing for vehicular communication. We compare the proposed key protocol with other related methods using Shamir secret sharing and the Chinese Remainder Theorem [11,17,20,21,22,28,29].

From Table 4, The proposed protocol satisfies many security features such as key authentication using symmetric HMAC protocol to ensure validity and prove the correct verification of the generated secret key. While the other mentioned protocols in [11,17,20,21,22,28,29] do not depend on symmetric authentication to verify the shared secret key, Liu et al. in [22] used the one-way hash function to satisfy the perfect secrecy of the key.

The proposed protocol also does not support the shares verification feature. The proposed key utilizes the concept of a trusted third party that is considered a trusted entity to generate and distribute secure secret shares to the participants’ vehicles. However, the fundamental Shamir secret in [11], Ulutas et al. in [17], and Liu et al. in [29] do not verify the generated shares that are considered a weak point and result in sharing fake shared secrets between participants. However, Liu et al. [29] support cheating detection by using two different polynomials and can detect cheaters up to k−1.

The proposed protocol and Harn et al. [20] do not require a secure channel between participants and the group manager or among participants themselves. For example, the participant vehicles in the proposed protocol do not communicate to reconstruct the master shared secret key, and all participants are receiving authenticated public points from the group manager to rebuild the secret key. However, the protocols mentioned in [11,17,21,22,28,29] require a secure broadcasting channel to allow the shareholders to participate in the key reconstruction process by sharing their shares.

Moreover, the proposed protocol is simple and unconditionally secure because it is based on polynomial interpolation and basic Shamir secret sharing protocol. The proposed protocol must satisfy two security conditions to be unconditionally secure. Firstly, any t or more than t shares allow the master secret S reconstruction; secondly, knowledge of any t shares cannot leak any information about the master secret. An attacker with t−1 public points cannot recover the secret key, and every participant needs at least t valid points over the polynomial to reconstruct the secret key. It is safe to say that the proposed key management protocol is 100% secure since it meets both of these criteria without making any computational assumptions. Shamir’s secret sharing technique is information-theoretically secure, which means that the arithmetic we examined is unbreakable even against an adversary with infinite computing power.

Computational security is built on computing assumptions that prevent attackers from solving mathematical issues due to their restricted computational capabilities. The prime factorization of a large integer number and the discrete logarithm solution are two examples of these mathematical difficulties. On the other hand, unconditional security suggests that the security can be assured even if no computational assumptions are used in the calculation. Shamir’s (*t*, *n*) protocol [11] is guaranteed to be secure at all times. It can meet the previously specified security criteria without relying on any computational assumptions.

The proposed protocol is based on Shamir secret sharing; however, the related works in [20,21,22,28] use different approaches for the shared secret key; in [20], Harn et al. use the Chinese Remainder Theorem (CRT). While in [21], Liu et al. use Asmuth–Bloom’s shared secret scheme, with additional improvements, Liu et al. in [22] use three different algorithms to satisfy the unconditional security and provide simplicity. In [22], the authors use a combination of Shamir secret protocol, generalized CRT theorem, and the Asmuth–Bloom technique. This combination offers perfect secrecy using a one-way hash function to verify the correctness of the secret.

We summarize the proposed protocol’s security features and other related shared secret protocols in Table 4.

From Table 4, we conclude the following:

- The proposed protocol outperforms the compared protocols [11,17,20,21,22,28,29] to support a lightweight symmetric authentication for the generated key that can be reconstructed securely at vehicles using the attached HMAC signature.
- In contrast to [11,17], the proposed protocol depends on centralized trusted third parties to satisfy the key security and support the key management process.
- The proposed protocol outperforms the other protocols to support key reconstruction without evolving the other participants in the key reconstruction process or ensuring the correctness of their shares. In contrast to the protocols in [20,21,22,28] that require adding of additional verification algorithm to verify the shared shares and prevent the fake shares from being broadcasted through the network.
- Thus, the proposed protocol reduces the computation cost and communicates cost to verify the participant shares by broadcasting authenticated public shares to allow all vehicles to reconstruct the master secret key.
- The proposed key management solution does not require a secure channel between participants to share their secret parts; thus, no additional overhead is required compared with the mentioned protocols [11,17,21,22,28,29].
- The proposed protocol is unconditionally secure compared with [20], requiring high computation security to support the same security functions.
- t or more than t are required to reconstruct the key in the proposed protocol using simplified polynomial interpolation and evaluations that makes the protocol suitable for critical vehicular applications that need fast verification at low-cost computation.
- The proposed protocol supports the key updates compared to the protocols in [11,17,20,21,22,28,29].
- According to shared policies, the group manager and the participant vehicles can agree on the periodic key update to prevent key compromise and sniffing attacks.

## 7. Performance Evaluation

This section introduces the computation and communication costs of the proposed protocol and some other linear shared secret protocols in [11,29].

### 7.1. Computation Overhead

For the comparison, we choose the linear shared secret protocols in [11,29] to compare the computation cost of the key generation and key reconstruction based on polynomial operations and Lagrange components.

In the proposed protocol, the G.M generates shares from a polynomial f(x) of degree t−1. Therefore, the polynomial has t coefficients, and t−1 is multiplied by x. The share generation represents the calculation of points over the polynomial f(x) of t−1 degree. The calculations over the finite field $F_{p}$ Each share generation requires one modulo addition operation and one modulo multiplication. The share generation overhead for the proposed protocol over the finite field $F_{p}$ is n(t−1) modular multiplication and nt modular addition operations.

The G.M chose t−1 points to publish in a broadcast way to all registered participants. The shared secret reconstruction at every participant requires the Lagrange interpolation operations to generate the Lagrange polynomials $l_{j}\left(x\right)$, then multiplying these polynomials with the t shares to reconstruct the original polynomial f(x) that is generated by the G.M previously.

We use this equation to reconstruct the secret key:

$$\prime f\left(x\right)=\sum_{j=1}^{t}\;y_{j}l_{j}\left(x\right)mod\left(p\right),\;\mathrm{where}\;l_{j}\left(x\right)=\prod_{i=1,i\neq j}^{t}\frac{x-x_{i}}{x_{j}-x_{i}}\;\;S=f\left(0\right)=\sum_{j=1}^{t}\;(y_{j}\;\prod_{i=1,i\neq j}^{t}\frac{x_{i}}{x_{i}-x_{j}})$$

The required operations for the key reconstruction are $\left(t^{3}+t+1\right)$ modular multiplication, t modular addition, and t modular multiplicative inverse.

The proposed protocol uses the basic Shamir secret sharing that enhances the computation cost. Compared to Liu et al. [29], combining two polynomials results in double computation cost for share generation and key reconstruction. Moreover, the proposed protocol and Shamir secret sharing have the exact computation cost due to the linearity of the used polynomial in both protocols.

Moreover, the proposed protocol uses the authentication of secret key based on HMAC protocol, generating HMAC signature can be negligible for both the G.M and the participant vehicles for small key sizes.

Table 5 summarizes the modular arithmetic operations over a finite field $F_{p}$ required to generate the secret shares for the proposed protocol and the linear (*t*, *n*) threshold protocols [11,29].

Table 6 summarizes the modular operation over the finite field $F_{p}$ to reconstruct the secret key at the participant side for the proposed protocol and the compared related work.

### 7.2. Communication Overhead

In the proposed protocol, only the G.M broadcasts a message m that consists of the following parameters: (t−1) public points $P_{i}$ on the polynomial f(x), for i=1;2; 3;…; t−1; a list of group members $n=\left\{V_{1},\;V_{2},\;V_{3},\;\ldots .,V_{n}\right\}$, the prim number p; and the threshold t attached to HMAC signature.

The G.M, during the initialization step, shares private information and one verified personal share with each vehicle through a private, secure channel. Therefore, the number of transmitted messages from the G.M in the proposed protocol is (n+1) message. In the proposed key generation protocol, the participants do not send any messages to each other. In contrast, the G.M sends one message to allow the group members to recover the group key and start secure communication.

The G.M’s messages are broadcast messages to allow the vehicles to recover the shared secret key. The communication cost is represented by the number of transmitted and received messages at the G.M and participant vehicles. The G.M in the proposed protocol sends one message in broadcast to allow the participant to recover the key. In contrast, the participant has to receive only one message from the G.M and send no messages for each other during the key reconstruction process.

The dealer in [11,29] has to share n messages for n participants during the key generation step, while in Liu et al. [29], the number of sent and received messages at vehicles is doubled compared with [11]. Each participant resends and receives two secret shares generated from two different polynomials.

In [11,29], the participants are transmitting their shares to t−1 shareholders to allow recovering the secret. Each participant requires other participants t−1 shares to recover the original secret key in addition to its share that was stored at the initialization phase. So, the sent messages from participants in [11,29] are (t−1), 2 (t−1) messages, respectively. In contrast, the received messages at each participant in [11,29] are t and 2t messages, respectively.

The number of transmitted messages at the participant vehicles for key reconstruction is zero for the proposed protocol. The participant does not have to communicate to collect other participants’ shares. The proposed protocol reduces the requirement of sending messages among participants that enhance the network performance and reduces the communication overhead cost. The number of received messages at each vehicle in the proposed protocol are two messages (one message from the group manager during the initialization phase and one message during the key reconstruction that the G.M broadcasted).

Table 7 summarizes the communication cost in terms of transmitted and received messages at both the G.M and the participants for the proposed protocol and [11,29].

For example, a threshold of (3–10) where 3≤t ≥10 and n=10, We calculated the transmitted messages from each participant, the received messages at each participant, and the total transmitted messages from the group manager (G.M).

**Figure 8:**
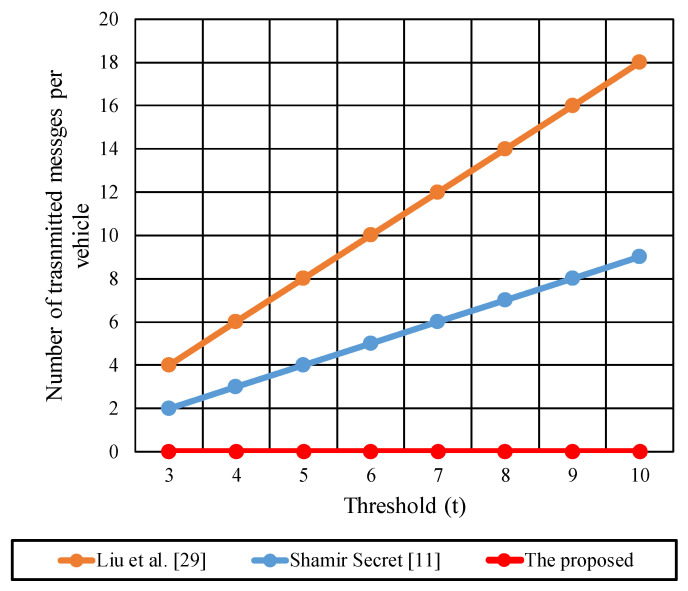
The transmitted messages per each participant vehicle during the key reconstruction phase.

Figure 8 shows the messages transmitted from each participant vehicle during the key reconstruction process for the (3–10) threshold protocols using Table 7 information.

Liu et al. [29] experience a linear increase in the number of transmitted messages corresponding to different t values where 3≤t ≥10 and n=10. The number of messages is doubled in [29] compared to [11] as Liu et al. are using two Shamir secret shares procedures that require the distribution of two secret shares per participant. The increase in t increases the transmitted messages and adds additional communication cost. In contrast to the proposed protocol, the participants do not share any messages that decrease the communication overhead and network congestion. Participants use the broadcasted messages from the G.M to recover the key in the proposed protocol. This reduces the need for secure channel establishment between participants’ vehicles.

**Figure 9:**
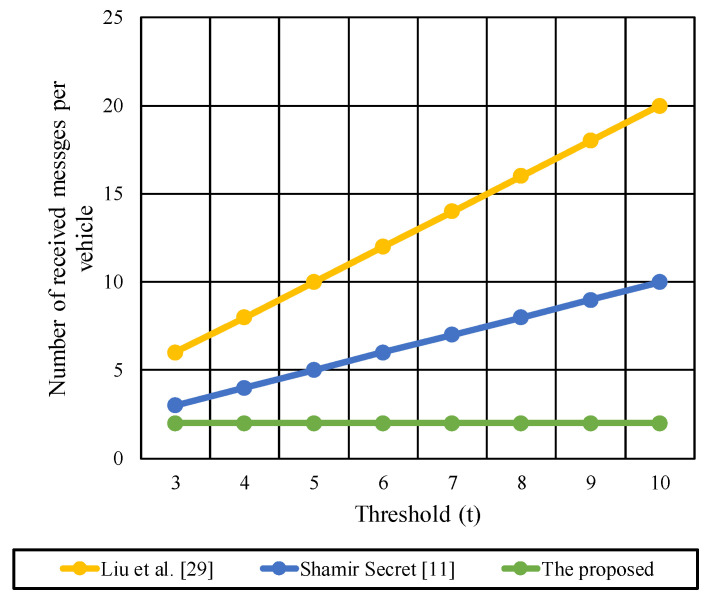
The received messages per participant vehicle during the key reconstruction phase.

Figure 9 shows the received messages per participant vehicle during the key reconstruction phase.

Each vehicle received t−1 message in the basic Shamir secret share [11] compared with  2(t−1)  in Liu et al. [29]. For the proposed protocol, each vehicle receives two messages from the G.M, one message during initialization and another message during the key reconstruction. The initialization message contains the vehicle’s secret part, while the second message contains the public secret shares from the G.M to start the key reconstruction process. The number of received messages is decreased from  2(t−1)  in Liu et al. to two messages in the proposed protocol. For large values of t, Liu et al. cannot be deployed due to the high communication cost at participant vehicles.

**Figure 10:**
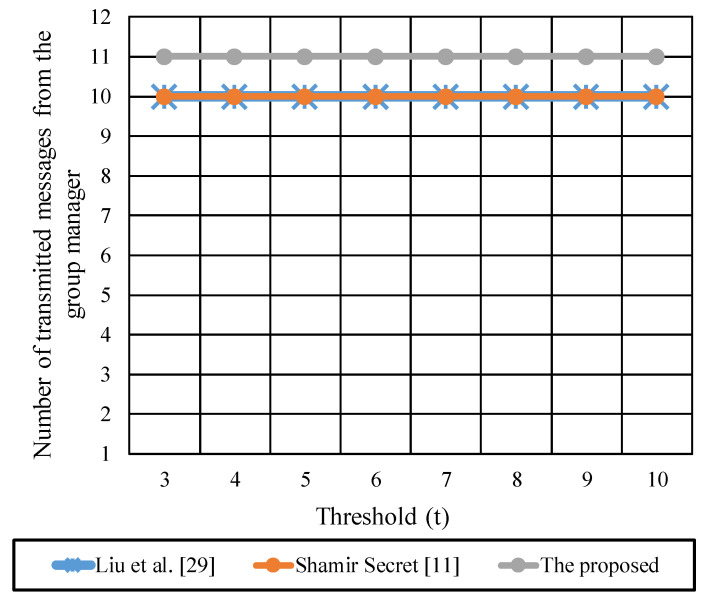
The total transmitted messages from the group manager during the key generation and reconstruction phases.

Figure 10 shows the total number of messages transmitted from the G.M (dealer) to the group participants during the key generation and key reconstruction. Both [11,29] are experienced total messages of  n  during the key generation and reconstruction phases. The G.M in the proposed protocol transmits  n+1  messages during the generation and construction of key. Where  n  messages are required to distribute the secret parts per participant through secure unicast channels, and one message is broadcasted to all participants during the reconstruction of the key. For *n* = 10, the transmitted messages in the proposed protocol are 11 messages, and in [11,29] are 10 messages. A slight difference between the compared protocol can be neglected to reduce the communication overhead between the participant.

We conclude from the above results that the proposed protocol reduces the transmitted messages overhead at vehicles from  2(t−1)  messages in previous shared secret protocols to zero messages. Moreover, it reduces the received messages at vehicles from 2t to two messages. The proposed centralized key generation protocol eliminates the requirement for secure channels between vehicles of the same group for the future key recovering process.

At the same time, it allows vehicles to store only a single secret share compared with other shared secret protocols that require the storage of t secret shares.

The proposed protocol is unconditionally secure and unbreakable using infinite computing power as t, or more than t secret shares to reconstruct the key. The proposed protocol security level outperforms the other shared secret protocols security, as it supports key authentication and confidentiality using HMAC that prevents attackers from compromising or faking the key.

## 8. Conclusions and Future Work

In this paper, we proposed a linear secret sharing protocol based on the Shamir secret method that allows one group manager to divide one secret key and distribute its parts to n participants. (t,n) participants are collaborating to recover the secret key and utilize it for future secure communication. Many shared secret protocols exist; however, they have some issues when applied in vehicular communication.

We proposed a shared secret protocol using linear polynomial interpolation and HMAC authentication for vehicular networks. The proposed protocol utilized the base stations as trusted third parties to generate secret shares and distribute the shares to all participants through a secure channel. It reduces the communication cost among participants to share their secret parts for the secret key recovering. The base station in the proposed protocol broadcasts some public information to allow the participants to reconstruct the group secret key. The participants recover the secret key using the public information from the base station and their secret parts from the key. Each participant needs at least t shares to recover the key. Less than t shares cannot reveal any information about the key, which makes the proposed protocol unconditionally secure.

The base station attaches an HMAC signature to each broadcasted message to allow the participants to authenticate the station and ensure the message’s integrity and authentication during transmission. The proposed protocol computation cost is lightweight and straightforward modular arithmetic operations over finite filed $F_{p}$ compared with the complex shared secret protocols using the discrete logarithm problems and elliptic curve mathematics. The proposed protocol has the same computation cost as the basic Shamir secret share [11]. Both protocols use the interpolation and evaluation of linear polynomials. However, the proposed protocol outperforms the basic Shamir secret sharing communication cost and security level. The proposed protocol reduces the transmitted messages overhead at vehicles from 2(t−1) messages in previous shared secret protocols to zero messages. Moreover, it reduces the received messages at vehicles from 2t to two messages. The proposed centralized key generation protocol eliminates the requirement for secure channels between vehicles of the same group for the future key recovering process.

The secret shares are not broadcast between vehicles, enhancing protocol security and resisting key recovery and compromising attacks.

Finally, the proposed protocol satisfies some security features such as key authentication, key confidentiality, linear polynomial interpolation, and unconditional security.

We intend to study the relation between t and n for different vehicular scenarios for future work. Moreover, we plan to integrate the polynomial-based shared secret and bilinear pairing over the elliptic curve to support key generation and data authentication.

## Figures and Tables

**Table 1:** The proposed protocol contributions over the basic Shamir secret protocol.

Security Properties	Basic Shamir Secret Sharing Issues	The Proposed Protocol Contributions
Misleading members	Assume all members of the group are honest. Individual members can mislead or ignore other participants’ contributions.	This assumption cannot be held in an untrustworthy and decentralized network. Members are not involved in the key reconstruction.
Decentralizing	Group members must communicate to exchange their secret parts required for key recovery. Require t participants to share their secret pieces to recover the original key.	Support centralized implantation where the base stations work as trusted group managers for vehicles. No need for the existence of t participants. The group manager broadcast t−1 public shares that are very important for only registered vehicles to recover the key.
High dynamic networks	It is impractical in V2X communication with high dynamic nature as many vehicles can join or leave the group frequently. At least t members must exist in the network.	Adding or removing group members does not affect the generated secret shares and the original key reconstruction. The group manager provides any required shares to reconstruct the key.
High communication overhead	Substantial overhead owing to high communication among participants.	No need for communication between participants to share their secret parts. Vehicles depend on the received information from the group manager.
Requirement of a secure channel	The communications between group members require secure channels.	No need for secure channels between participants. We are reducing the overhead of establishing secure channels between vehicles.
Key recovery attacks	Vulnerable to key recovery attacks since each participant’s secret part is broadcasted in a broadcast channel.	Resistance to key recovery attacks by reducing the communication between vehicles at the initialization phase.
The misbehaving dealer	If the dealer is a bad actor, the private key can be stolen or abused.	The dealer in the proposed protocol is trusted and authenticates itself via HMAC authentication protocol.
The key authentication and confidentiality	It’s a security issue since the private key must first be produced and divided into parts.	The proposed protocol supports the key authentication using HMAC signatures.
Verification of secret shares	Requires the verification of secret shares to ensure the correctness of shared secret parts.	Only authorized and registered vehicles can join the network. No need for secret shares verification process. Vehicles are not required to broadcast their secret parts to other vehicles.

**Table 2:** Comparison of the previous shared secret protocols in terms of advantages and disadvantages.

Shared Secret Protocol	Advantages	Disadvantages	Implementation Type
Basic Shamir [11]	No verification of secret shares. Unconditionally secure. (*t* − *n*) threshold protocol.	Require secure channel. Require group members’ communication. High communication cost. No key updating and authentication.	Decentralized
Ulutas et al. [17]	Secure distribution of shares of medical images. No verification of secret shares. (*t* − *n*) threshold protocol.	No key updating. No key authentication. Dishonest and fake shares distribution.	Decentralized
Harn et al. [20]	Support a verifiable secret sharing method based on the CRT. Using Asmuth–Bloom’s scheme. It does not require a secure channel.	Require verification of secret shares. High communication cost. Computationally secure. No key updating and key authentication.	Centralized
Liu et al. [21]	Efficient secret share using Asmuth–Bloom’s scheme. Unconditionally secure.	Require verification of secret shares. No key updating and no key authentication. Require secure channel.	Centralized
Liu et al. [22]	Unconditionally secure. Using Chinese Reminder Theorem, Shamir’s Secret Sharing, and Asmuth-secret Bloom’s sharing. Support key authentication using a one-way hash function.	Require verification of secret shares. Require secure channel. No key updating. High communication cost.	Centralized
Meng et al. [28]	Presented two thresholds changeable secret sharing methods. Using a mix of bivariate and univariate polynomials. Unconditionally secure.	Require secure channel. Require verification of secret shares. No key updating and authentication.	Centralized
Liu et al. [29]	A linear threshold secret sharing that combines two of Shamir’s procedures. Cheating detection. No verification of secret shares.	No key updating and key authentication. High communication cost due to using of two polynomials.	Centralized

**Table 3:** The generated secret shares by the trusted G.M over the polynomial $f\left(x\right)=12+10x+20x^{2}$.

$x_{i}$	1	2	3	4	5	6	7	8	9	10	…
$y_{i}$ = $f\left(x_{i}\right)$	19	20	15	4	10	10	4	15	20	19	…

**Table 4:** Security features comparison for the proposed key management protocol and other shared secret related works.

Security Features	Shamir Secret [11]	Ulutas et al. [17]	Harn et al. [20]	Liu et al. [21]	Liu et al. [22]	Meng et al. [28]	Liu et al. [29]	The Proposed
Authentication using HMAC	No	No	No	No	No	No	No	Yes
Key updating	No	No	No	No	No	No	No	Yes
Verification of secret shares	No	No	Yes	Yes	Yes	Yes	No	No
Secure channel requirement	Yes	Yes	No	Yes	Yes	Yes	Yes	No
Centralized implementation	No	No	Yes	Yes	Yes	Yes	Yes	Yes
Based Shamir Secret Share	Yes	Yes	No	No	Yes	No	Yes	Yes
(t,n) secret sharing	Yes	Yes	Yes	Yes	Yes	Yes	Yes	Yes
Group member broadcasting	Yes	Yes	Yes	Yes	No	Yes	Yes	No
Unconditionally secure	Yes	Yes	No	Yes	Yes	Yes	Yes	Yes

**Table 5:** The computation cost of shares generation for the proposed protocol and some linear shared secret protocols.

The Shared Secret Protocols	Modular Arithmetic Operations over a Finite Field *F_p_*		
	Modular Multiplication	Modular Addition	Modular Multiplicative Inverse
Shamir Secret [11]	n(t−1)	nt	-
Liu et al. [29]	2n(t−1)	2nt	-
The proposed	n(t−1)	nt	-

**Table 6:** The computation cost of share reconstruction for the proposed protocol and some linear shared secret protocols.

The Shared Secret Protocols	Modular Arithmetic Operations over a Finite Field *F_p_*		
	Modular Multiplication	Modular Addition	Modular Multiplicative Inverse
Shamir Secret [11]	$\left(t^{3}+t+1\right)$	t	t
Liu et al. [29]	$2\left(t^{3}+t+1\right)$	2t	2t
The proposed	$\left(t^{3}+t+1\right)$	t	t

**Table 7:** The communication cost for the proposed protocol and some other related shared secret protocols.

The Shared Secret Protocols	Sent Messages from G.M (Dealer) during Key Reconstruction	Sent Messages from Participants Vehicles	Received Messages at Participants Vehicles
Shamir Secret [11]	n	t−1	t
Liu et al. [29]	n	2(t−1)	2(t)
The proposed	n+1	-	2

Note: t represents the required secret shares to recover the key.
